# Characterization of the broad-spectrum antibacterial activity of bacteriocin-like inhibitory substance-producing probiotics isolated from fermented foods

**DOI:** 10.1186/s12866-024-03245-0

**Published:** 2024-03-11

**Authors:** Tran Thi Dieu Thuy, Hsu-Feng Lu, Carl Jay Ballena Bregente, Fong-Chi Annabelle Huang, Pei-Chun Tu, Cheng-Yen Kao

**Affiliations:** 1 Institute of Microbiology and Immunology, College of Life Sciences, National Yang Ming Chiao Tung University, No. 155, Sec. 2, Linong Street, Taipei, 112 Taiwan; 2 Department of Laboratory Medicine, China Medical University Hospital, Taichung, Taiwan; 3 Department of Medical Laboratory Science and Biotechnology, Asia University, Taichung, Taiwan; 4 College of Medical Technology, Southwestern University PHINMA, Cebu, Philippines; 5 Taipei American School, Taipei, Taiwan; 6 Health Innovation Center, National Yang Ming Chiao Tung University, Taipei, Taiwan; 7 Microbiota Research Center, National Yang Ming Chiao Tung University, Taipei, Taiwan

**Keywords:** Antibacterial activity, Bacteriocin-like inhibitory substance, Biofilm formation, Multidrug-resistant bacteria, Probiotic

## Abstract

Antimicrobial peptides, such as bacteriocin, produced by probiotics have become a promising novel class of therapeutic agents for treating infectious diseases. Selected lactic acid bacteria (LAB) isolated from fermented foods with probiotic potential were evaluated for various tests, including exopolysaccharide production, antibiotic susceptibility, acid and bile tolerance, antibacterial activity, and cell adhesion and cytotoxicity to gastric cell lines. Six selected LAB strains maintained their high viability under gastrointestinal conditions, produced high exopolysaccharides, showed no or less cytotoxicity, and adhered successfully to gastric cells. Furthermore, three strains, *Weissella confusa* CYLB30, *Lactiplantibacillus plantarum* CYLB47, and *Limosilactobacillus fermentum* CYLB55, demonstrated a strong antibacterial effect against drug-resistant *Escherichia coli, Klebsiella pneumoniae*, *Pseudomonas aeruginosa*, *Salmonella enterica* serovar Choleraesuis, *Enterococcus faecium*, and *Staphylococcus aureus.* Whole genome sequencing was performed on these three strains using the Nanopore platform; then, the results showed that all three strains did not harbor genes related to toxins, superantigens, and acquired antimicrobial resistance, in their genome. The bacteriocin gene cluster was found in CYLB47 genome, but not in CYLB30 and CYLB55 genomes. In SDS-PAGE, the extract of CYLB30 and CYLB47 bacteriocin-like inhibitory substance (BLIS) yielded a single band with a size of less than 10 kDa. These BLIS inhibited the growth and biofilm formation of drug-resistant *P. aeruginosa* and methicillin-resistant *S. aureus* (MRSA), causing membrane disruption and inhibiting adhesion ability to human skin HaCaT cells. Moreover, CYLB30 and CYLB47 BLIS rescued the larvae after being infected with *P. aeruginosa* and MRSA infections. In conclusion, CYLB30 and CYLB47 BLIS may be potential alternative treatment for multidrug-resistant bacteria infections.

**Supplementary Information:**

The online version contains supplementary material available at 10.1186/s12866-024-03245-0.

## Background

Antimicrobial resistance (AMR) is a serious global health concern that causes significant illness and high mortality of patients infected with multidrug-resistant (MDR) bacteria [1]. Therefore, the investigation of new antimicrobial drugs or alternative therapies capable of overcoming AMR is urgent. Antimicrobial peptides (AMPs), derived from probiotics or humans, are promising alternative antimicrobial agents to treat MDR bacteria and biofilm-related infections due to their effective antibacterial action without developing resistance [2].

Currently, the gastric acid- and bile salt-tolerant lactic acid bacteria (LAB) are the most widely used probiotics in humans because these bacteria have many benefits for humans, such as immune response modulation, gastrointestinal adsorption, microbiota homeostasis, and antipathogenic activity [3]. LAB use fermentation as a natural bioprocessing technique to produce foods such as milk, veggies, yogurt, cheese, and meat, which contributes to enhancing the nutritional value and maintaining the quality of food and beverage items for a long time [4]. Many studies have demonstrated that a bactericidal mode of action is always present in biologically active peptides or protein complexes, such as small AMPs (bacteriocins) generated by LAB, and also in other closely related species of gram-positive bacteria [5].

AMPs derived from probiotics are typically effective against foodborne pathogenic bacteria, including *Bacillus cereus*, *Clostridium perfringens*, *Staphylococcus aureus*, and *Listeria monocytogenes* [5]. Most LAB bacteriocins act by disturbing the cytoplasmic membrane through forming pores, or by degradation of the cell wall [6]. Gram-positive bacteria that produce bacteriocins are categorized into three classes according to their size, structure, and modifications [6, 7]. Class I and II consist of low molecular weight bacteriocins (≤ 10 kD) and are heat-stable, but class I bacteriocins have post-translational modification characterized by the presence of unusual amino acids, while class II bacteriocins do not undergo post-translational modification. Class III consists of all high molecular weight bacteriocins (10 kD) [6, 7].

Therefore, bacteriocins have been proposed to be beneficial antibiotic substitutes because they possess significant potency, high stability, low toxicity, and wide and narrow activity spectra that make them appropriate for medical applications [8]. In this study, our objective was to isolate and characterize bacteriocin-like inhibitory substance (BLIS)-producing probiotics from fermentation foods with potential applications against clinically MDR pathogens, particularly *Escherichia coli, Klebsiella pneumoniae*, *Pseudomonas aeruginosa*, *Salmonella enterica* serovar Choleraesuis, *Enterococcus faecium*, and *S. aureus*, by using phenotypic tests and whole genome sequencing.

## Results

### Isolation and characterization of LAB from fermented foods

To select strains with potential applications, we initially used LAB-specific primers to screen LAB strains, determined the growth of strains in MRS broth, and tested their ability to produce EPS. Among the 32 strains isolated from fermented foods purchased from various traditional markets in Taiwan, only 11 of them were LAB. Six LAB strains, including CYLB30, CYLB33, CYLB45, CYLB47, CYLB51, and CYLB55, produced high EPS, ranging from 355.5 to 380.1 mg/mL (Table S1), and grew well in MRS broth (Fig. S1), were selected for further investigations. The growth rate of six selected strains in MRS broth was slower than the SLC13 control strain (Fig. S1). The results of 16s rDNA sequencing showed that CYLB30, CYLB33, CYLB45, CYLB47, CYLB51, and CYLB55, were *Weissella confusa*, *Pediococcus stilesii*, *Lactobacillus futsaii*, *Lactiplantibacillus plantarum*, *Weissella confusa*, and *Limosilactobacillus fermentum*, respectively (Table S1).

The antibiotic susceptibility of six LAB strains to 12 antimicrobial agents was examined, and the results showed that all strains were resistant to ciprofloxacin, gentamicin, levofloxacin, and metronidazole (Table S1). However, they were susceptible to ampicillin, amoxicillin/clavulanate, ceftriaxone, penicillin G, tetracyclines, and clarithromycin (Table S1). *L. plantarum* CYLB47 was susceptible to amikacin but resistant to cefazolin, while the others were resistant to amikacin but susceptible to cefazolin (Table S1).

We further determined the survival of LAB under mimic simulated gastrointestinal conditions in vitro, and the results revealed that 5 of 6 strains, CYLB30, CYLB33, CYLB45, CYLB47, and CYLB55, showed high tolerance to low pH and 0.3% bile salt (Fig. S2). In addition, although CYLB51 was not resistant to low pH, losing 46.5% survival after 3 h exposure, it was tolerant to 0.3% bile salt and maintained 113.5% survival after 4 h incubation (Fig. S2).

### Adhesion ability and cytotoxicity of LAB to AGS, GES-1, and Caco-2 cells

**Fig. 1:**
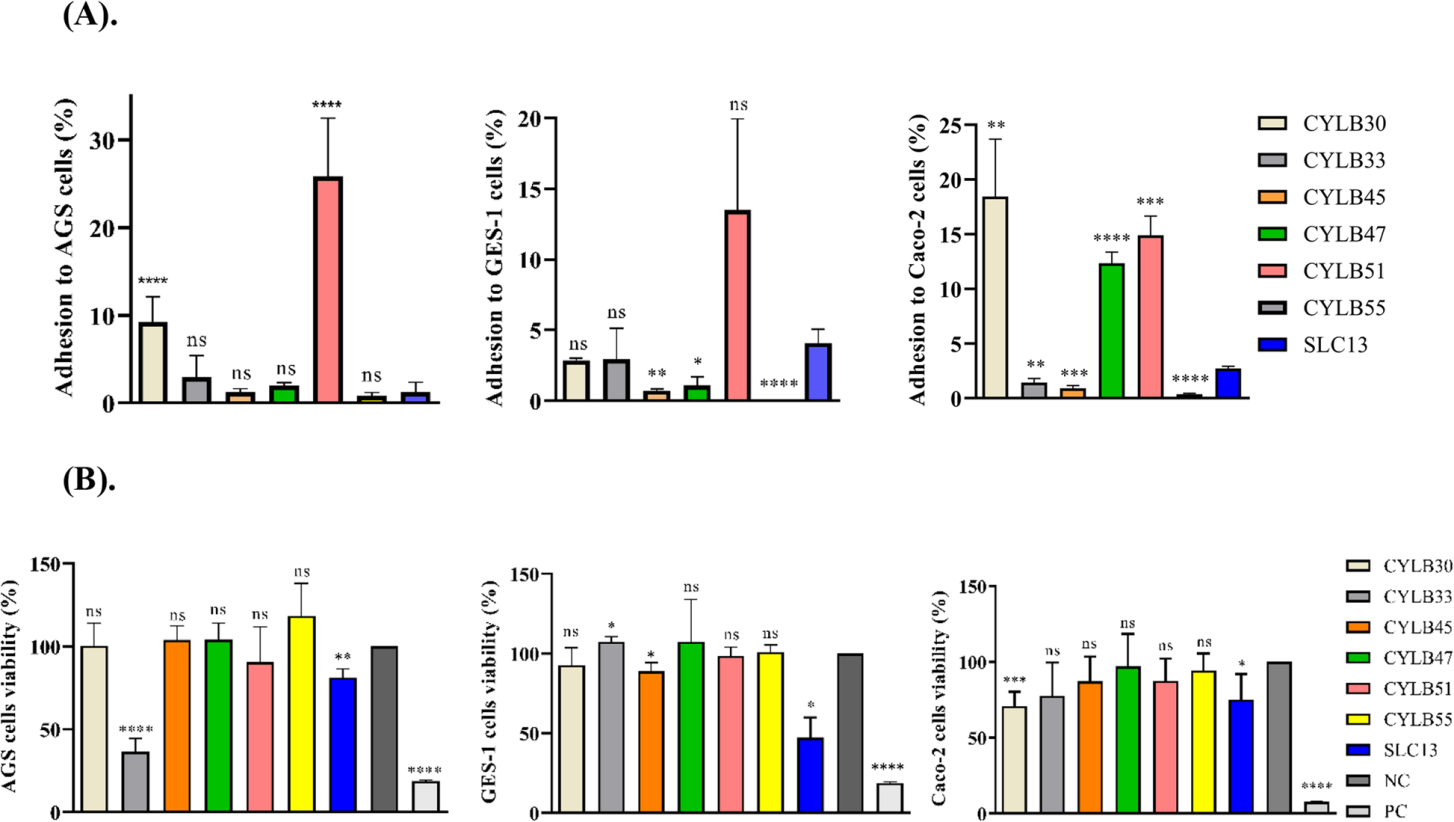
Cell adhesion ability and cytotoxicity of LAB strains.** A **Adhesion of isolated LAB strains to AGS, GES-1, and Caco-2 cells with MOI=100 for 90 min. The cell adhesion of the LAB isolated from this study was compared to SLC13.** B** Cell viability of AGS, GES-1, and Caco-2 cells treated with MOI=100 of LAB for 24 h. Cells treated with 0.1% Triton X-100 served as a positive control (PC). The cell cytotoxicity of LAB isolated from this study was compared to the negative control (NC, medium only). The error bars represent the standard deviation of triplicates. *, *p* < 0.05; **, *p* < 0.01; ***, *p* < 0.001; ****, *p* < 0.0001; ns, no significant difference

The adhesion rates of CYLB30, CYLB33, CYLB45, CYLB47, CYLB51, and CYLB55 to AGS, GES-1, and Caco-2 cells altered depending on the strains tested, which ranged from 0.84 to 25.8%, 0.05 to 13.5%, and 0.34 to 19.8%, respectively (Fig. 1A). CYLB51 showed the highest adhesion rates, by 25.8% and 13.5%, to AGS and GES-1 cells, respectively, while CYLB30 showed the highest adhesion rate (18.4%) to Caco-2 cells (Fig. 1A).

Cell viability assay was conducted to evaluate the cytotoxicity of LAB strains on AGS, GES-1, and Caco-2 cells (Fig. 1B). Cells treated with 0.1% Triton X-100 significantly inhibited cell proliferation and therefore served as a positive control in this assay (Fig. 1B). The results indicated that CYLB33 (36.6% survival rate) and SLC13 (81.3% survival rate) had cytotoxicity to AGS cells, CYLB45 (88.8% survival rate) and SLC13 (47.3% survival rate) had cytotoxicity to GES-1 cells, and CYLB30 (70.9% survival rate) and SLC13 (75.1% survival rate) had significant cytotoxicity to Caco-2 cells, at MOI 100 (Fig. 1B). Overall, CYLB47, CYLB51, and CYLB55, did not show significant cytotoxicity on three cell lines (Fig. 1B).

### Antibacterial activity of LAB against pathogens

Six multidrug-resistant pathogenic bacteria (four gram-negative and two gram-positive bacteria) were selected to evaluate the antibacterial activity of isolated LAB strains by using overlay assay. The results showed that only three strains, CYLB30, CYLB47, and CYLB55, had inhibitory activity against all pathogens tested. Moreover, CYLB30 and CYLB47 showed the strongest inhibition activity against pathogens compared to other strains (Table S2).

### Genome analysis of CYLB30, CYLB47, and CYLB55

We further performed whole genome sequencing on three strains that had higher antibacterial activity against all specified pathogenic bacteria. The reads were assembled, and the contigs returned with the head segment were almost identical to the tail segment, indicating the circular nature of the contigs. The results of Nanopore genome sequencing showed that the CYLB30 genome comprises a 2,410,104-bp chromosome and six plasmids with a size range from 10,284 to 25,018-bp; CYLB47 genome comprises a 3,157,658-bp chromosome and nine plasmids with a size range from 11,629 to 71,871-bp; CYLB55 genome comprises a 2,093,653-bp chromosome and two plasmids with a size of 8,672 and 38,056 bp (Table S3). Importantly, based on the results of RAST annotation, no genes classified as “Toxin and Superantigens” and “Virulence, Disease, and Defense” were identified in CYLB30, CYLB47, and CYLB55. Additionally, no acquired antimicrobial resistance genes were detected in the three strains by using ResFinder.

### Bacteriocin gene cluster analysis and extraction

**Fig. 2:**
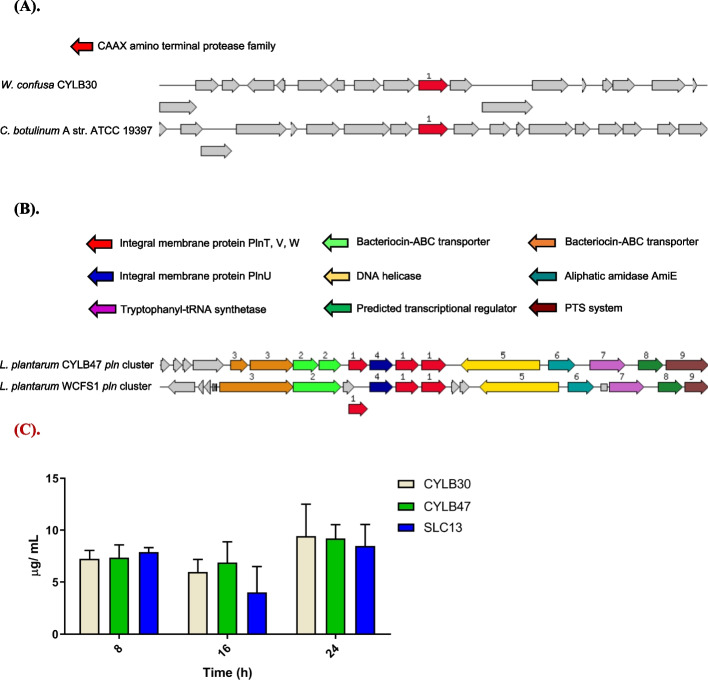
Genetic organization of genes for bacteriocin-like inhibitory substance synthesis and its extraction. **A** Genetic organization of bacteriocin gene cluster of *W. confusa* CYLB30 and *C. botulinum* A str. ATCC 19397. **B** Genetic organization of *pln *gene cluster of *L. plantarum *CYLB47 and WCFS1. The graphic is centered on the focus gene (bacteriocin genes), which is red and numbered 1. Genes with similar sequences are clustered together and assigned the same number and color. The arrows were colored to represent various functions of the predicted genes. **C** Determination of the BLIS production in CYLB30, CYLB47, and SLC13 at different time points (8, 16, 24 hours) post-cultivation. The results indicate that the BLIS levels in these three strains show a slight decrease at 16 hours compared to 8 hours, but reach the highest production after 24 hours of cultivation

CYLB55 did not carry bacteriocin genes. A CAXX amino terminal protease family protein (intramembrane metalloproteases with various functions commonly present in bacteriocin gene clusters) was detected in CYLB30 (Fig. 2A). CYLB47 had a bacteriocin gene cluster which organization is similar to the cluster in *L. plantarum* WCFS1, including *plnT*, *plnU*, *plnV*, and *plnW* (Fig. 2B). We further utilized BAGEL to verify the presence of BLIS synthesis genes in CYLB30, CYLB47, and CYLB55. Consistent with the results in Fig. 2B, we identified the bacteriocin protein in the chromosome of CYLB47 as a garvicin Q family class II bacteriocin (Fig. S3). This protein is composed of 57 amino acids, and BLAST results indicate that the BLIS sequence in CYLB47 is identical to those previously identified in *Limosilactobacillus reuteri* DSM 20016, *Lacticaseibacillus hulanensis*, *Lactiplantibacillus pentosus*, *Furfurilactobacillus rossiae* DSM 15814, and *Lactiplantibacillus plantarum* strains. However, the BLIS synthesis gene in CYLB30 was not found in the BAGEL analysis. These results may be attributed to the relatively limited BLIS sequence data available for *Weissella confusa*. We further extracted BLIS from CYLB30 and CYLB47 and used SDS-PAGE to validate the crude extract of BLIS (Fig. S4). No BLIS was detected in CYLB55 and this observation was consistent with our WGS data; however, BLIS with molecular weight less than 10 kDa was detected in CYLB30, CYLB47, and SLC13 (positive control) (Fig. S4). Moreover, the results indicate that the BLIS levels in the culture medium of these three strains show a slight decrease at 16 h compared to 8 h, but reach the highest production after 24 h of cultivation (Fig. 2C).

### Antibacterial activity of CYLB30 and CYLB47 bacteriocin-like inhibitory substance

**Fig. 3:**
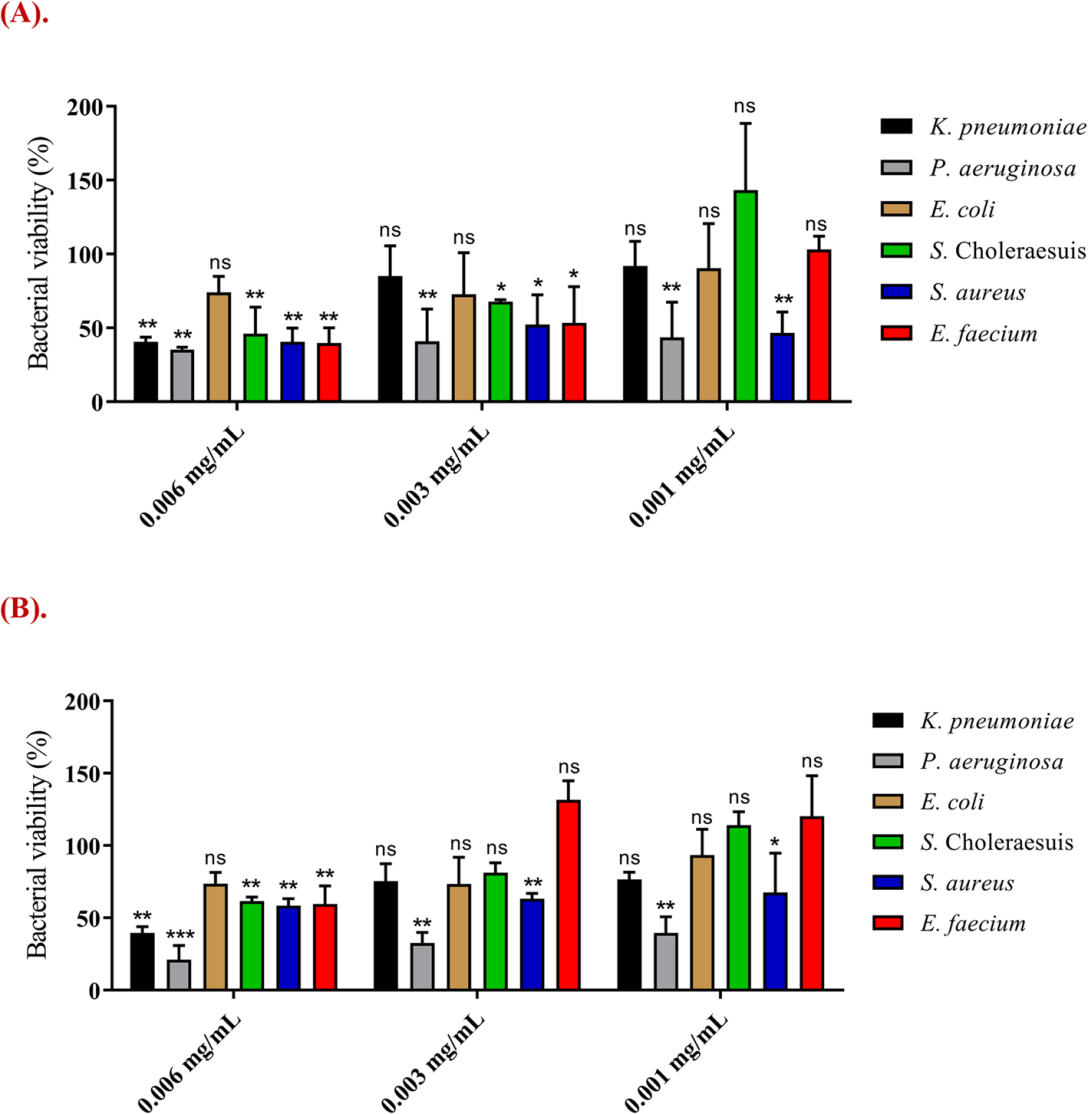
Antibacterial activity of bacteriocin-like inhibitory substance (BLIS) extracted from CYLB30 (**A**) and CYLB47 (**B**). Six pathogenic bacteria, including *Salmonella enterica* serovar Choleraesuis, *E. coli*, *K. pneumoniae*, *P. aeruginosa*, *E. faecium*,and *S. aureus*, were treated with BLIS. Error bars represent the standard deviation of triplicates. The bacterial viability of pathogens was compared to a negative control without BLIS treatment. *, *p* < 0.05; **, *p* < 0.01; ***, *p* < 0.001; ****, *p* < 0.0001; ns, no significant difference

We further determined the antibacterial activity of extracted BLIS against six selected pathogens, and the results revealed that CYLB30 and CYLB47 BLIS inhibited the growth of five of the six selected pathogenic bacteria (*K. pneumoniae*, *Salmonella enterica* serovar Choleraesuis, *P. aeruginosa*, *E. faecium*, and *S. aureus*) with the highest concentration that we can obtain optimally (final concentration: 0.006 mg/mL) (Fig. 3A and B). Moreover, the data revealed that these two BLIS strongly inhibited pathogens in a dose-dependent manner (Fig. 3).

### Inhibition of biofilm formation by bacteriocin-like inhibitory substance

**Fig. 4:**
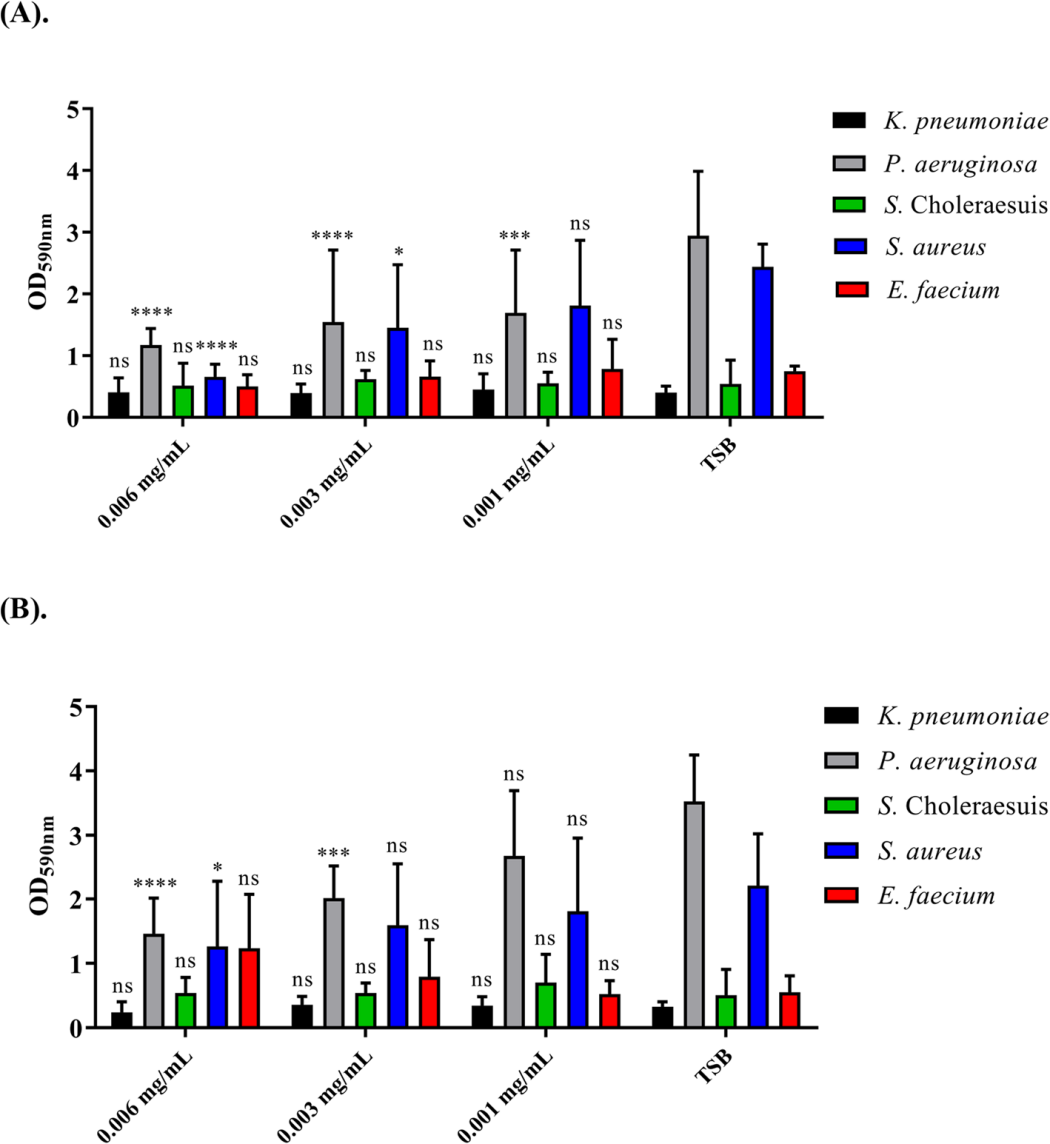
The anti-biofilm activity of bacteriocin-like inhibitory substance (BLIS) extracted from CYLB30 (**A**) and CYLB47 (**B**). Five pathogenic bacteria, including *Salmonella* *enterica* serovar Choleraesuis, *K. pneumoniae*, *P. aeruginosa*, *E. faecium*, and *S. aureus*, were treated with BLIS. After crystal violet staining, the biofilm was dissolved in absolute ethanol and measured by OD_590nm_. Error bars represent the standard deviation of triplicates. The biofilm formation of pathogens was compared to a negative control without BLIS treatment (TSB group). *, *p* < 0.05; ***, *p* < 0.001; ****, *p* < 0.0001; ns, no significant difference

Biofilm-related infections are difficult to treat and antibiotic monotherapy is often insufficient, even though certain rediscovered classical drugs have demonstrated remarkable efficacy. Therefore, we next investigated the effect of BLIS on the biofilm formation of pathogenic bacteria (Fig. 4). *E. coli* was excluded from this test due to the low antibacterial activity of BLIS shown in Fig. 3. Serial concentrations of BLIS were exposed to five pathogenic bacteria cultures for 24 h in a 96-well plate; then, biofilm formation was examined by crystal violet staining. In treatment with the highest final concentration of CYLB30 BLIS (0.006 mg/mL), the biofilm formation of *P. aeruginosa* and *S. aureus* decreased significantly compared to the control without BLIS treatments (Fig. 4A). The BLIS from CYLB47 showed similar effects on biofilm formation of *P. aeruginosa* and *S. aureus* at high concentrations (0.006 mg/mL) (Fig. 4B). Moreover, the data showed that these two BLIS inhibited the biofilm formation of pathogens in a dose-dependent manner (Fig. 4). The antimicrobial activity of probiotics may be attributed to BLIS, hydrogen peroxide (H_2_O_2_), and lactic acid production. Therefore, we conducted an analysis focusing on the most BLIS-sensitive strain of *P. aeruginosa*. We attempted to assess the antimicrobial activity using the CFS of CYLB30 and CYLB47, including neutralized-CFS, CFS pretreated with proteinase K, and CFS pretreated with catalase (Fig. S5). The results revealed that neutralized CFS lost its antimicrobial activity, indicating that acidic components may play a role in the observed inhibition. Interestingly, proteinase K treatment did not impact the antimicrobial activity. Furthermore, catalase treatment only resulted in a decrease in the antimicrobial activity of CYLB47’s CFS (Fig. S5B).

### Bacteriocin-like inhibitory substance cause membrane permeability of pathogens

**Fig. 5:**
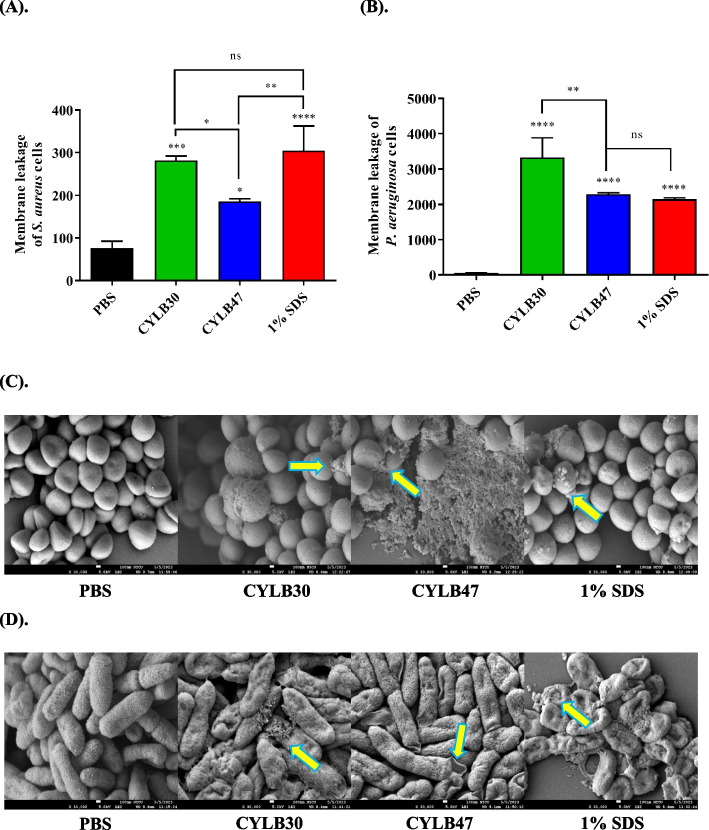
Effects of bacteriocin-like inhibitory substance (BLIS) on bacteria membrane permeability of *S. aureus *(**A**) and *P. aeruginosa *(**B**) by PI staining, and the morphology of *S. aureus *(**C**) and *P. aeruginosa *(**D**) observedby SEM analysis after 24 h treatments. PBS and 1% SDS were considered negative and positive control, respectively, for PI staining. The yellow arrows indicate intracellular debris released from cells or damaged cells due to the pore formation in bacterial cell walls. Error bars represent the standard deviation of triplicates. *, *p* < 0.05; ***, *p* < 0.001; ****, *p* < 0.0001; ns, no significant difference

To evaluate the antibacterial mechanisms of the BLIS tested, we performed PI staining after the treatment of pathogens, including gram-positive *S. aureus* and gram-negative *P. aeruginosa*, with BLIS at 0.006 mg/mL for 24 h. The results proved that membrane disruption occurred when these BLIS were added to pathogens, allowing the fluorescence dye to bind to the nucleic acid of *S. aureus* and *P. aeruginosa*, resulting in high fluorescence intensity (Fig. 5A and B). Furthermore, when pathogens were treated with the same concentration of BLIS, CYLB30 BLIS caused more severe damage to the membrane of *S. aureus* and *P. aeruginosa* than CYLB47 BLIS. Surprisingly, CYLB30 BLIS was even more effective than 1% SDS at causing membrane leakage of the *P. aeruginosa* model (Fig. 5B) and was equally effective as in 1% SDS in the *S. aureus* model (Fig. 5A).

Furthermore, we used SEM to observe the bacterial cell morphology after exposure to 0.006 mg/mL of CYLB30 and CYLB47 BLIS for 24 h (Fig. 5C and D). Untreated *S. aureus* and *P. aeruginosa* cells were intact with smooth cell walls. However, after BLIS treatments, cells became shriveled, apertures formed in the cell wall, and intracellular debris was discharged, indicating that the cell membranes of *S. aureus* and *P. aeruginosa* had been damaged (Fig. 5C and D).

### Anti-adhesion activity of bacteriocin-like inhibitory substance

**Fig. 6:**
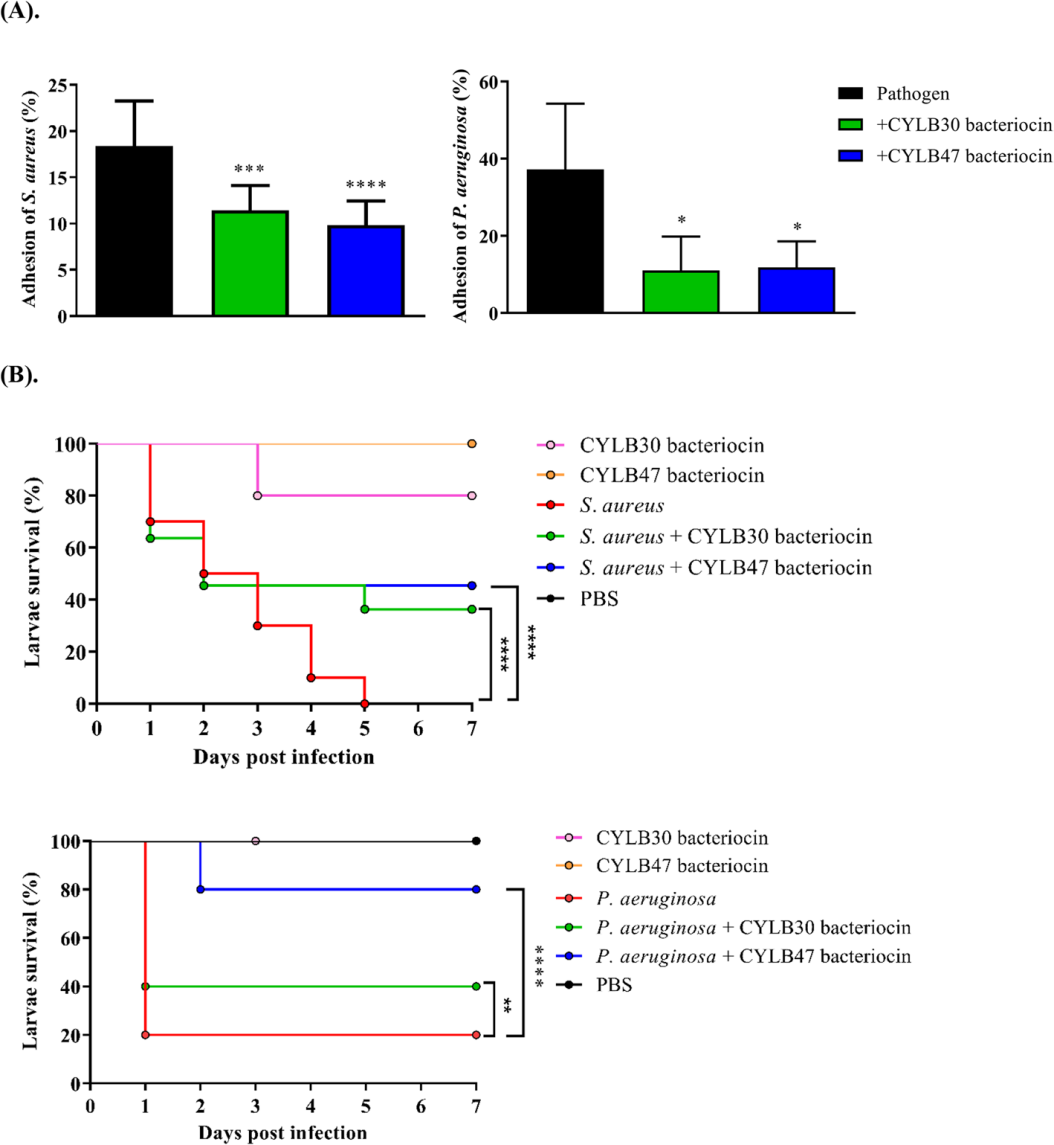
The effect of CYLB30 and CYLB47 BLIS on the virulence of *S. aureus *and *P. aeruginosa *by examining their adhesion ability to HaCaT cells (**A**) and the survival of *Galleria mellonella *larvae (**B**). **A **The adhesion rate was determined by counting colony formation after exposure to *S. aureus *or *P. aeruginosa *for 2 h incubationwith or without BLIS treatments. **B **The survival percentage of larvae was observed for 7 days after injection of *S. aureus* or *P. aeruginosa* with or without BLIS treatments. Ten larvae were used under each experimental condition, and the experiment was repeated three times. Error bars represent the standard deviation of triplicates. *, *p* < 0.05; ***, *p* < 0.001; ****, *p* < 0.0001; ns, no significant difference

*S. aureus* and *P. aeruginosa* adhere efficiently to human tissues, particularly the skin, causing infections that are difficult to treat [9, 10]. Following the findings that CYLB30 and CYLB47 had the ability to inhibit these two pathogens by reducing biofilm formation and causing membrane damage, we sought to investigate the effect of BLIS on the adhesion ability of two pathogens to human skin HaCaT cells. The results showed that treatment with CYLB30 and CYLB47 BLIS effectively reduced the adhesion rate of *S. aureus* and *P. aeruginosa* to cells compared to the untreated group (Fig. 6A).

### *Galleria mellonella* larvae infection model to evaluate the antibacterial activity of bacteriocin-like inhibitory substance

We investigated the survivability of *G. mellonella* larvae treated with CYLB30 and CYLB47 BLIS and *G. mellonella* larvae infected with *S. aureus* and *P. aeruginosa* with and without BLIS treatment. The larvae were injected with 10 µL of bacteria culture or PBS control in the second to last proleg. The injected larvae were observed every 24 h for seven days to assess their survival rate. The *G. mellonella* larvae were injected with BLIS after 12 h infected with *S. aureus* or *P. aeruginosa* and incubated at 37 °C for up to 7 days. As shown in Fig. 6B, CYLB30 and CYLB47 BLIS did not show toxicity to cause a significant decrease in larvae survival. After BLIS treatment for *S. aureus* infection, larvae survival rates were significantly rescued and maintained at around 35–45% after seven days compared to *S. aureus* infection without treatments, larvae were all dead after five days of infection (Fig. 6B). CYLB30 and CYLB47 BLIS demonstrated similar efficacy in reducing the virulence of *S. aureus* to larvae. Similarly, with *P. aeruginosa* infection, BLIS treatment can improve the larvae survival rate after seven days compared to *P. aeruginosa* infection without treatment. Interestingly, CYLB47 BLIS showed a more significant protective effect compared to CYLB47 BLIS, which showed 80% survival of larvae after seven days of infection (Fig. 6B).

## Discussion

In recent years, researchers have focused more on utilizing natural polymers in various fields, which has resulted in the advancement of research on EPS-producing LAB. EPS have been used in the food industry as viscosity, stabilizing, and emulsifying agents, as well as having potential health advantages such as antioxidant, anticancer, anti-inflammatory, and antiviral activity [11, 12]. LAB have captured the attention of researchers due to their exceptional capacity to create EPS. The previous study found that bacteria produced high EPS and increased tolerance to harsh environments such as acid and bile salt [13]. Our results are consistent with the previous study; the selected six strains that produced high EPS showed resistance to gastrointestinal conditions (Table S1 & Fig. S2). However, the biological benefits of EPS produced from strains isolated in this study remain unclear. The adhesion rates of isolates from human feces were reported to be much higher compared to the LAB isolated from fermented food [14]. However, this study found that our LAB isolated from fermented foods adhered to human cells at significantly high rates, especially strain *W. confusa* CYLB51 (Fig. 1A). Based on these results, we can further investigate how these bacteria adapt and colonize successfully to human cells and figure out the effect of these strains on the host’s immune response for further applications.

LAB can inhibit or kill pathogens through various mechanisms, including organic acids production, competitive exclusion, immune system modulation, and AMPs such as BLIS production [14, 15]. Regarding strains with high antibacterial activity, we performed whole genome sequencing to identify the potential gene cluster responsible for BLIS biosynthesis and its broad-spectrum antimicrobial activity. These strains studied did not show any virulence, toxin, or acquired antimicrobial resistance genes; it can therefore be established that the bacteria are safe for use in the food industry with antibacterial activity against foodborne pathogens. Although *L. fermentum* CYLB55 did not harbor any genes related to BLIS biosynthesis, this strain showed high antibacterial activity against pathogens (Table S2). Therefore, these results suggest that the mechanism(s) through which CYLB55 inhibits all pathogens tested is due to the production of organic acids and other factors. Furthermore, many reports mentioned that *L. fermentum* could enhance the immunologic response and have the ability to decrease the level of bloodstream cholesterol (as cholesterol-lowering agents), thus mediately inhibiting pathogens [16].

Multiple methods have been used to screen for BLIS production in vitro, including the soft overlay agar assay (test producer strains are pipetted in small volumes onto the surface of agar plates, which are overlayed with soft agar containing the indicator strain) [17] and the well-diffusion assay (using whole bacteria or, more commonly, cell-free supernatants) [18]. However, the limitations of these methods are that they can not differentiate between the inhibitory activity of BLIS or other antimicrobial substances, such as organic acids and phenol-soluble modulins [19]. Therefore, we combined WGS and BLIS extraction to validate the production and antibacterial activity of BLIS. Although SDS-PAGE is not an accurate technique to determine the small molecular mass, our results showed a single band for extracted BLIS from CYLB30, CYLB47, and SLC13 (Fig. 2C). Interestingly, we could not find the gene cluster for BLIS synthesis in CYLB30 from the WGS results. These results suggest that the unknown gene cluster may contribute to the novel BLIS production and is worth investigating.

Our WGS results further validated that *L. plantarum* CYLB47 produced known plantaricin, which is an integral membrane protein. Previous studies showed that BLIS belonged to class II with a small size (< 10 kDa) produced by *L. plantarum* is known as plantaricin with a variety of bactericidal/bacteriostatic mechanisms [20]. Regarding these, CYLB47 plantaricin might target *S. aureus* and *P. aeruginosa* via the mode of action of lantibiotics (type A lantibiotics kill rapidly by pore formation or type B lantibiotics inhibit peptidoglycan biosynthesis [21]). The results of SEM observation were consistent with our WGS analysis, CYLB47 plantaricin displayed pore formation ability on the *S. aureus* and *P. aeruginosa* under SEM observation (Fig. 5C and D). However, there were few sufficient studies on BLIS from *Weissella* spp., especially *W. confusa*. A previous survey showed that *W. confusa* A3 BLIS was isolated from fermented milk belonging to class II BLIS with stability after exposure to low pH, pepsin, and heat [22] and was shown to have inhibitory activity toward pathogenic bacteria, namely *B. cereus*, *E. coli*, *P. aeruginosa* and *Micrococcus luteus* [22]. We also identified that our *W. confusa* CYLB30 BLIS showed strong antibacterial activity against *S. aureus* and *P. aeruginosa in vitro* and in vivo (Figs. 3 and 6). Combined with the SEM observation and PI staining, this evidence indicated that the CYLB30 BLIS could also contribute to disrupting the membrane and resulted in a reduction of *S. aureus* and *P. aeruginosa*. Although these findings suggest that acidic components released by probiotics are the primary contributors to antimicrobial activity, the question of whether proteinase K can completely inhibit the BLIS produced by CYLB30 and CYLB47, as well as the activity of BLIS in a neutral but not in the acidic environment, remain to be clarified [23]. Additionally, our results demonstrate that the CFS from CYLB30 and CYLB47 can induce membrane leakage in bacteria (Fig. 5). Therefore, the antibacterial activity of BLIS contained within the CYLB30 and CYLB47 CFS is worthy investigating.

Biofilm-related infections account for up to 80% of human microbiological diseases and are related to common human illnesses such as diabetes, poor oral hygiene, and medical implants [24]. Although a lower dose of CYLB47 plantaricin decreased the survival of *S. aureus*, this BLIS could not lead to the reduction of *S. aureus* biofilm formation (Figs. 3 and 4). These results suggest the protective role of biofilms against small amounts of AMPs. The larvae of *G. mellonella* have previously been used as a model host for studying the virulence of pathogenic microbes, including *S. aureus* [25, 26] and *P. aeruginosa* [27, 28]. The larvae have also been used to study the efficacy of various antimicrobial agents. A previous study found that epidermicin is a novel AMP with potent activity against MRSA. When administered 2 h post-infection at a maximum dose of 200 mg/kg, epidermicin significantly increased survival in larvae after five days of observation compared to MRSA alone [26]. In our MRSA infection model, CYLB30 and CYLB47 BLIS treatments showed similar impacts with epidermicin on the reduction of *S. aureus* virulence. They rescued around 35–40% larvae survival after seven days compared to *S. aureus* alone (Fig. 6B). However, the mode of action and characteristics of CYLB30 and CYLB47 BLIS remain to be investigated for future applications.

Furthermore, the other AMPs isolated from the rumen microbiome were assessed for their therapeutic potential against seven clinical strains of *P. aeruginosa*. According to their data, those compounds decreased the virulence of *P. aeruginosa* and maintained 50% of the larvae survival after five days with the same dose of *P. aeruginosa* [28]. Our results showed that both CYLB30 and CYLB47 BLIS had significant inhibition of *P. aeruginosa* virulence in larvae (Fig. 6B). In particular, CYLB47 plantaricin exhibited better efficiency compared to the CYLB30 BLIS in in vivo larvae model. Based on SEM data, the CYLB30 and CYLB47 BLIS shared a similar mode of action to target the bacteria membrane. However, in addition to that mode of action, these two BLIS probably had other mechanisms to inhibit pathogens, which will be further validated for the subsequent study.

## Conclusions

CYLB30 and CYLB47 BLIS showed considerable antibacterial efficacy, anti-biofilm formation, and anti-adhesion against MRSA and MDR *P. aeruginosa*, as well as the pathogenicity of these pathogens on larvae survival. The CYLB30 and CYLB47 BLIS killed the bacteria by damaging their outer membrane, and these BLIS were both safe and effective. To treat illnesses caused by antibiotic-resistant bacteria infections, new antibiotics and therapies are desperately needed. Therefore, CYLB30 and CYLB47 BLIS have the potential to be effective and safe antibacterial agents in clinical practice but need further characterization.

## Methods

### Bacterial strains and cultivation conditions

The bacterial strains used in this study are listed in Table S1. We previously isolated a high exopolysaccharide (EPS)-producing *Lactiplantibacillus pentosus* strain, SLC13, which showed high antibacterial activity [29], and was used as the control strain in this study. Fermented food samples, including kimchi, mustard pickle, and stinky tofu for LAB isolation, were collected from various traditional markets in Northern Taiwan. The samples were transported to the laboratory at room temperature for isolation within 24 h. The liquid of each sample was mixed with 1x phosphate-buffered saline (PBS). A serial dilution was performed and colonies formed on MRS agar plates (de Man, Rogosa, and Sharpe) (Wonwon Biotechnology, Taiwan) after 48 h at 37 °C. Colonies with different colony morphologies were randomly picked from MRS agar plates, and then the selected colonies were initially tested by PCR with LAB-specific primers. The LAB strains were stored at − 80 °C in MRS broth containing 16% glycerol until testing.

Six pathogenic bacteria listed in Table S1 were maintained on tryptic soy agar (TSA) plates (Wonwon Biotechnology, Taiwan) and routinely cultured in tryptic soy broth (TSB) at 37 °C. In addition, those strains were stored at − 80 °C in TSB containing 20% glycerol until testing.

### DNA extraction, LAB identification, and 16s rRNA sequencing

DNA extraction from gram-positive bacteria was performed according to the previous study [30]. Putative LAB strains isolated from fermented foods were verified by PCR using LAB-specific primers [31]. The sequence of the 16s rDNA gene was determined for species identification. Primers and conditions for PCR amplification of the 16s rDNA were previously described [32]. The 1,500-bp PCR products were sequenced and compared with the known 16s rDNA gene sequences deposited in NCBI GenBank for species identification.

### Exopolysaccharide extraction

EPS was extracted and determined based on a previous study with minor modifications [14]. The LAB strains were inoculated in 25 mL MRS broth containing 2% sucrose at 37 ^o^C for 24 h. Bacterial cultures were centrifuged at 4,000 xg for 15 min to remove cells and debris. EPS precipitation was extracted by adding a 4-fold volume of 95% ethanol, and the mixture was incubated at 4 °C for 24 h. After ethanol precipitation, the samples were centrifugated at 4,000 xg for 15 min, and the precipitate of crude EPS was dried in the oven at 60 °C for 24 h. The precipitates were resuspended in distilled water and filtered with 0.45 μm diameter filters. EPS production was analyzed by phenol-sulfuric method using glucose as a reference standard [33].

### Cell culture and adhesion assay

AGS cells (human gastric adenocarcinoma epithelial cells, ATCC CRL 1739), GES-1 cells (normal human gastric epithelial cells), Caco-2 cells (human colorectal adenocarcinoma cells), and HaCaT cells (immortalized human keratinocytes) were maintained in Ham′s F12, RPMI 1640, DMEM, and DMEM medium, respectively, supplemented with 10% fetal bovine serum (FBS) (Gibco BRL, Life Technologies, Rockville, MD), penicillin (100 IU/mL), and streptomycin (100 μm/mL) at 37 °C with 5% CO_2_, respectively. AGS and GES-1 cells were a kind gift from Professor Hsiu-Chi Cheng (National Cheng Kung University). Caco-2 and HaCaT cells were a gift from Professor Wei-Lun Hwang (National Yang Ming Chiao Tung University) and Professor Yi-Hsien Hsieh (Chung Shan Medical University), respectively.

To determine the adhesion ability of LAB, cells (3 × 10^5^) were seeded into 12-well plates in media supplemented with 10% FBS and 1% penicillin-streptomycin and grown to a monolayer at 37 °C for 24 h. Cells were cocultured with LAB at a multiplicity of infection (MOI) of 100 and incubated at 37 °C for 90 min. Cells were washed three times with PBS, lysed by incubation with 0.1% saponin (for AGS and GES-1 cells) or 0.1% Triton X-100 (for Caco-2 and HaCaT cells) at 37 °C for 15 min, and then plated on MRS agar plates. Colonies were counted to determine the number of bacteria that adhere to the cells. The percentage of bacterial adhesion to cells was calculated according to the following formula: adhesion ability (%) = (adherent bacteria)/(total bacteria) x 100. Cell adhesion assay was conducted in biological triplicate to ensure reproducibility.

### LAB cytotoxicity on AGS, GES-1, and Caco-2 cells

The cytotoxicity effect of LAB on AGS, GES-1, and Caco-2 cells, was determined by 3-(4,5-dimethylthiazol-2-yl)-2,5-diphenyltetrazolium bromide assay (MTT; Sigma-Aldrich, St Louis, MO, USA) according to the previous study [34]. Cells (3 × 10^5^) were grown and allowed to adhere to a 96-well plate at 37 °C for 48 h to approximately 80% confluence, and then cells were treated with LAB strains at a MOI of 100 for 24 h. The medium was removed, and the cells were washed three times with PBS. Thirty µL of MTT reagent (0.05 mg/mL) was added to each well, and the plates were incubated at 37 °C for 3 h in the dark. Two hundred µL of DMSO was added to each well, and the plate was incubated with shaking (150 rpm) at room temperature for 15 min to allow the color to develop. The optical density (OD) was measured at 570 nm. Cell viability (%) = (A_sample_/A_control_)×100, where A_sample_ is the absorbance of the cells incubated with the medium containing LAB or 0.1% Triton X-100, and A_control_ is the absorbance of the cells alone. The cytotoxicity assay was conducted in biological triplicate to ensure reproducibility.

### Antibacterial activity determined by overlay assay

LAB were cultured on MRS agar plates at 37℃ for 24 h; then, the single colony was picked up by 200 µL tip and spotted on MRS agar plates. These plates were incubated at 37℃ for 6 h. Selected pathogens (*E. coli*, *K. pneumoniae*, *P. aeruginosa*, *Salmonella enterica* serovar Choleraesuis, *E. faecium*, and *S. aureus*, Table S1) were cultured in 5 mL TSB overnight at 37℃. One mL of overnight cultured bacteria was co-incubated with 9 mL 0.8% agar (dissolved in TSB), poured onto MRS plates, and became overlaid. These plates were incubated at 37℃ for 24 h, and the inhibition zone was measured to assess the antibacterial activity of LAB.

### Genome sequencing, assembly, annotation, and analysis

The genomic DNA (gDNA) of CYLB30, CYLB47, and CYLB55 was extracted using a Presto™ gDNA Bacteria Advanced Kit (Geneaid Biotech, Ltd., Taiwan) from a 10 mL MRS broth bacterial culture, followed by the protocol for gram-positive bacteria. The whole genome sequence of LAB was determined by Nanopore genome sequencer. One µg gDNA was used to construct the sequencing library using a Ligation Sequencing Kit (SQK-LSK109, Oxford Nanopore Technologies, Oxford, UK). KAPA HyperPure (Oxford Nanopore Technologies, Oxford, UK) was used to purify the gDNA fragments. The Oxford Nanopore MinION MK1C was used to determine the whole genome sequence of three strains. A total of 300 ng gDNA was loaded onto the R9.4.1 flow cell. The quality of reads generated was assessed using Fast QC v0.11.5 (https://www.bioinformatics.babraham.ac.uk/projects/fastqc/). The raw signals were translated into a DNA sequence using ONT Gussy basecalling program (version 4.2.3). These genomes were constructed with Flye *de novo* assembler (version 2.9), and the options for backward compatibility (plasmids) and uneven coverage mode (meta) were used in this analysis [35, 36]. Genome sequences were deposited in the NCBI GenBank database.

ResFinder (http://www.genomicepidemiology.org/) was used to find the antibiotic resistance genes. Genome annotation was performed by NCBI Prokaryotic Genome Annotation Pipeline (PGAP, version 6.1) (https://www.ncbi.nlm.nih.gov/genome/annotation_prok/). We also used Rapid Annotations using Subsystems Technology (RAST, https://rast.nmpdr.org/rast.cgi) to predict the subsystem of genes and identify BLIS gene cluster in genomes (annotation scheme, classic RAST, gene caller, RAST; FIGfam version, Release70; automatically fix errors and frameshifts). The amino acid sequences of the BLIS gene cluster were further used as a query in a Psi-Blast search at the NCBI to obtain similarities to known genes and a summary of the recognized domains in the gene product. BAGEL4 (http://bagel5.molgenrug.nl/) [37] was further used to identify and visualize gene clusters involved in the biosynthesis of Ribosomally synthesized and Post translationally modified Peptides (RiPPs) and (unmodified) bacteriocins in CYLB30, CYLB47, and CYLB55.

### Bacteriocin-like inhibitory substance extraction and molecular weight estimation by SDS-PAGE

LAB strains were cultured in 100 mL MRS broth at 37 ℃ for 8 h, 16 and 24 h. The culture of LAB strains was centrifuged (4,000 ×g, 10 min) and BLIS extraction was precipitated from the supernatant by adding 2.6 M ammonium sulfate [(NH_4_) _2_SO_4_] at a 1:2 ratio (v/v) at 4 °C overnight [38]. Subsequently, the precipitate was separated from the filtrate by centrifugation under cooling at 13,000 xg for 10 min, and the residue was dissolved in 1x PBS buffer. Although we did not desalt the extracted bacteriocin using chromatography, we initially employed an Amicon ultra centrifugal filter (3 K molecular weight cutoff, Sigma) with PBS buffer washing for desalting. Subsequently, we used a 10 K molecular weight cutoff Amicon ultra centrifugal filter to remove > 10 KDa substance and repeat the antimicrobial assay. The final precipitation was dissolved in 20 mM citrate buffer at pH 5.5. The dissolved precipitate was used for the BLIS activity test, while the rest was stored at -20 °C until tested.

The concentration of protein was determined by Bradford assay standard curve of concentration versus absorbance. The concentration of protein (in mg/mL) was determined using the equation y = 0.0329x + 0.2757 with an R^2^ value of 0.9903. The molecular weight of BLIS was analyzed and estimated using SDS-PAGE [39]. The gel used for the separation was 16.5% Tris-Tricine SDS-PAGE, and the ladder used was PageRuler™ Prestained Protein Ladder (Thermo Scientific™) with a range of 10–180 kDa. The gel was subjected to 100 mV for 2 h and then stained with Coomassie blue (Thermo Scientific™) for BLIS visualization.

### Bacteriocin-like inhibitory substance antibacterial assay

Six drug-resistant pathogens were used (Table S1), including *E. coli*, *K. pneumoniae*, *Salmonella enterica* serovar Choleraesuis, *P. aeruginosa*, *E. faecium*, and *S. aureus*, to test the antibacterial activity of BLIS. The concentration of pathogens with a value of 0.2 at OD_600nm_ was mixed with the final BLIS concentrations as serial concentrations, which were 0.006, 0.003, and 0.001 mg/mL in a total volume of 200 µL in TSB at 37 °C for 24 h. Pathogen survival was calculated according to the number of colonies grown on TSA plates, compared to the number of pathogens without BLIS treatments. Pathogenic bacteria survival rate (%) = [D_1_(Log CFU (colony-forming unit)/mL)/D_2_(Log CFU/mL)] × 100, where D_1_ is the viable count of pathogenic bacteria with BLIS treatment for 24 h, and D_2_ is the viable count of pathogenic bacteria without treatments after 24 h. Each strain was analyzed in triplicate wells on at least three separate occasions.

### Biofilm formation determined by crystal violet staining

Tested pathogens were cultured in TSB overnight. A total of 200 µL of TSB containing serial final concentrations of BLIS (0.006, 0.003, and 0.001 mg/mL) was dispensed into sterile 96-well microplates and inoculated with an overnight culture of each pathogenic strain to give a final inoculum of 1% and incubated for 24 h at 37 °C. The supernatants were removed, and the biofilms formed on the bottoms of the wells were washed twice with PBS. The biofilms were fixed with methanol for 30 min at room temperature. After removing the supernatants, the biofilms were stained with 0.1% crystal violet for 15 min, washed twice with PBS, and resuspended in 200 µL of absolute ethanol. Biofilm formation was quantified by measuring the OD at 590 nm after crystal violet staining.

### Anti-adhesion activity of bacteriocin-like inhibitory substance

HaCaT cells (3 × 10^5^ cells) were seeded into 12-well plates in DMEM medium supplemented with 10% FBS and 1% penicillin-streptomycin and grown to a monolayer at 37 °C for 24 h. To determine the anti-adhesion ability of extracted BLIS, cells were infected with an MOI of 100 of *S. aureus* or *P. aeruginosa* and exposed to 0.006 mg/mL (final concentration) of extracted BLIS, then incubated at 37 °C for 2 h. Cells were washed three times with PBS, lysed by incubation with 0.1% Trion X-100 at 37 °C for 15 min, and plated on TSA plates. Colonies were counted to determine the number of adherent bacteria. The percentage of bacterial adhesion to cells was calculated according to the following formula: adhesion ability (%) = (adherent bacteria)/(total bacteria) x 100. Cell adhesion assay was conducted in biological triplicate to ensure reproducibility.

### Membrane permeability tests

BLIS shearing the membrane of the target bacteria can cause the bacterial DNA to be released and then bound with to fluorescent dye. Hence, the higher fluorescence indicates that BLIS was more effective in inhibitory activity on target bacteria. The membrane permeability test was performed using propidium iodide (PI) dye following the method used in a previous study with modifications [40]. The final concentration of *S. aureus* or *P. aeruginosa* was fixed at OD_600nm_ of one in 1x PBS, then exposed to BLIS (final concentration: 0.006 mg/mL) and incubated at 37 °C for 24 h. The mixture was centrifuged at 13,000 xg for 10 min and resuspended in 0.5 mL of PBS. Subsequently, 2 µL of PI was added to the bacterial suspension, followed by incubation at room temperature for 5 min. The treated samples were detected by fluorescence microscopy (PI: OD 535/617 nm, 200x magnification). *S. aureus* or *P. aeruginosa* was cultured at 37 °C for 24 h in PBS alone or treated with 1% SDS as control, and each treatment was performed in triplicate.

### Scanning electron microscopy observation

Scanning electron microscopy (SEM) was performed as a report of Reichhardt et al. with minor modifications [41]. *S. aureus* or *P. aeruginosa* was cultured at 37℃ for 16 h, then the bacterial cells were collected by centrifugation at 13,000 xg for 10 min. The final concentration of bacteria in PBS was fixed at OD_600nm_ of one in PBS, then exposed to the extract BLIS (final concentration: 0.006 mg/mL) and incubated at 37 °C for 24 h. Treatment with 1% SDS and PBS were considered positive and negative controls, respectively. After treatment, cells were harvested by centrifugation at 13,000 xg for 10 min and washed twice with 1x PBS. The samples were fixed overnight in 2.5% (v/v) glutaraldehyde at 4 °C and washed twice with 1x PBS. Cell pellets were dehydrated in a series of graded ethanol (10%, 30%, 50%, 70%, and 90%) for 10 min each and resuspended in 50 µL of 100% ethanol. Then, 20 µL of the mixture was transferred to a sterile 8 mm coverslip in a 24-well plate and allowed to dry for 5–7 min. Finally, 500 µL of 100% ethanol was soaked in each well. After ion spray gold coating, bacteria morphology was observed by ultra-high resolution thermal field emission SEM (JSM-7600 F FESEM (JEOL, Ltd., Tokyo, Japan).

### Antibacterial activity of bacteriocin-like inhibitory substance examined by *Galleria mellonella* larvae model

*Galleria mellonella* larvae model was used to evaluate the safety and the antibacterial activity of BLIS on the virulence of *S. aureus* and *P. aeruginosa in vivo* [42]. Larvae weighing between 200 mg and 240 mg were used in the assays. Briefly, *S. aureus* and *P. aeruginosa* were harvested, dissolved into 3 mL of PBS, and diluted to 10^8^ CFU/mL and 10^4^ CFU/mL, respectively. A Hamilton syringe was used to inject 10 µL aliquots of diluted bacteria into the left proleg of larvae. After a 6 h infection by pathogenic bacteria, *G. mellonella* larvae were injected with 10 µL of 0.006 mg/mL of BLIS. The CFU of bacterial suspension was evaluated by bacterial CFU on TSA plates at 37℃ for 24 h. As a check against any potential fatal consequences of the injection or incubation processes, 10 µL of PBS was used as a negative control, and 10 µL of 0.006 mg/mL of BLIS, were injected into larvae to access safety. Ten *G. mellonella* larvae were used under each experimental condition, and the procedure was repeated at least three times. The survival of larvae was monitored every 24 h for 96 h after injection while they were incubated in Petri dishes at 37 °C under normal aerobic conditions. The larvae were deemed dead when they did not respond to light poking with a pipette tip [43].

### Statistical analysis

Statistical analyses were performed using GraphPad Prism software (version 8.0.2, USA). The mean and standard deviation are used to express all the results. Tukey’s method with one-way analysis of variance (ANOVA) was utilized to compare the difference between groups. Log-rank (Mantel-Cox) test was performed to compare the survival of larvae. A *p* value *<* 0.05 was considered significant difference.

### Supplementary Information

**Supplementary Material 1.**

## Data Availability

Whole genome sequencing data are available in the NCBI database under BioProject accession PRJNA945178 (CYLB30), PRJNA945330 (CYLB47), PRJNA945331 (CYLB55); and BioSample accession SAMN33770553 (CYLB30), SAMN33777523 (CYLB47), SAMN33777524 (CYLB55). Complete genome sequences of CYLB30, CYLB47, and CYLB55 strains have been deposited in GenBank under the accession numbers CP120514-CP120520 (CYLB30), CP120655-CP120667 (CYLB47), and CP120668-CP120670 (CYLB55). The other data will be available on request.
