# Supplementary material for: Characterization of the broad-spectrum antibacterial activity of bacteriocin-like inhibitory substance-producing probiotics isolated from fermented foods
Source: BMC Microbiol. 2024 Mar 11;24:85. doi: 10.1186/s12866-024-03245-0 (PMC10926564; doi:10.1186/s12866-024-03245-0)
Supplement: Supplementary file 1 — Supplementary Material 1. [file 12866_2024_3245_MOESM1_ESM.docx]

**Materials and methods**

**Growth curve of LAB strains**

The growth rate of LAB was determined over 24 h at 37°C using a BioTek Synergy HTX multimode reader. Briefly, LAB was cultured in 10 mL MRS broth at 37°C for 24 h. Then 200 μL of MRS broth (pH=6.5) was dispensed into sterile 96-well microplates and inoculated with an overnight culture of each strain to obtain a ﬁnal inoculum of 1%. Readings were taken every 15 min of absorbance of each well (scanned at OD_600nm_) in the microplates for 24 h.

S. aureus or P. aeruginosa was cultured at 37 °C for 24 h in PBS alone or treated with 1% SDS as control, and each treatment was performed in triplicate.

**Antibiotic susceptibility tests**

Antibiotic susceptibility was determined through disk diffusion assay against 12 antibiotics (amikacin, amoxicillin/clavulanate, ampicillin, cefazolin, ceftriaxone, ciprofloxacin, clarithromycin, gentamicin, levofloxacin, metronidazole, penicillin G, and tetracycline) on MRS agar plates using BD BBL Sensi-Discs (Becton Dickin­son, Sparks, MD) according to the Clinical and Laboratory Standards Institute (CLSI) guidelines. E. coli ATCC 25922 was used as a quality control strain. All measurements were performed in triplicate and the interpretation of resistance to these antimicrobial agents was determined according to the recommendations of the CLSI (2022).

**Tolerance to simulated gastrointestinal conditions**

To test the survival of isolated LAB strains in acidic environment (pH=3.0) or in bile salt condition (0.3%), the bacteria were incubated at 37°C. The viable organisms were counted after exposure to acid or bile salt conditions for 3 h or 4 h on MRS agar incubated at 37^o^C for 48 h [1]. Survival cell count was calculated according to the number of colonies grown on MRS agar, compared to the initial bacterial concentration.

LAB survival rate (%) = [A_1_(Log CFU/mL)/A_0_(Log CFU/mL)]x100, where A_0_ is the viable count of LAB at 0 h, and A_1_ is the viable count of LAB after 3 h incubation in acidic MRS broth (pH=3.0). Each strain was analyzed in triplicate wells on at least three separate occasions.

LAB survival rate (%) = [B_1_(Log CFU/mL)/B_0_(Log CFU/mL)] × 100, where B_0_ is the viable count of LABs at 0 h, and B_1_ is the viable count of LABs after 4 h incubation in MRS broth with 0.3% bile salt. Each strain was analyzed in triplicate wells on at least three separate occasions.

**Genome sequencing, assembly, annotation, and analysis**

Genomic DNA (gDNA) of CYLB30, CYLB47, and CYLB55 was extracted using a Presto™ gDNA Bacteria Advanced Kit (Geneaid Biotech, Ltd., Taiwan) from a 10 mL MRS broth bacterial culture, followed by the protocol for Gram-positive bacteria. The whole genome sequence of LAB was determined by Nanopore genome sequencer. One μg gDNA was used to construct the sequencing library using a Ligation Sequencing Kit (SQK-LSK109, Oxford Nanopore Technologies, Oxford, UK). KAPA HyperPure (Oxford Nanopore Technologies, Oxford, UK) was used to purify the gDNA fragments. The Oxford Nanopore MinION MK1C was used to determine the whole genome sequence of three strains. A total of 300 ng gDNA was loaded onto the R9.4.1 flow cell. The quality of reads generated was assessed using Fast QC v0.11.5 (https://www.bioinformatics.babraham.ac.uk/projects/fastqc/). The raw signals were translated into a DNA sequence using ONT Gussy basecalling program (version 4.2.3). These genomes were constructed with Flye *de novo* assembler (version 2.9), and the options for backward compatibility (plasmids) and uneven coverage mode (meta) were used in this analysis [2, 3]. Genome annotation was performed by NCBI Prokaryotic Genome Annotation Pipeline (PGAP, version 6.1) (https://www.ncbi.nlm.nih.gov/genome/annotation_prok/). BAGEL4 (http://bagel5.molgenrug.nl/) [4] was used to identify and visualize gene clusters involved in the biosynthesis of Ribosomally synthesized and Post translationally modified Peptides (RiPPs) and (unmodified) bacteriocins in CYLB30, CYLB47, and CYLB55.

**Bacteriocin-like inhibitory substance antibacterial assay**

To analyze the inhibitory effect of the cell-free supernatant (CFS) from CYLB30 and CYLB47 strains on the growth of *Pseudomonas aeruginosa* after pH adjustment, proteinase K, and catalase treatment, we adjusted the CFS pH to 6.5 using NaOH. Additionally, we pretreated the CFS with either 3 mg/mL proteinase K (incubated for 1 hour at 37 ℃) or 3 mg/mL catalase enzyme from bovine liver (incubated for 1 hour at 37 ℃). These treated-CFS samples were then added to 96-well microplates. The microplates were inoculated with an overnight culture of *P. aeruginosa*, resulting in a final inoculum of 1%, and incubated for 24 hours at 37°C. Each well had a total volume of 200 μL, with 100 μL of treated-CFS and 100 μL of bacterial culture. Absorbance readings were recorded at 15-minute intervals for 24 hours, measuring the OD_600_ nm for each well in the microplates.

**Table S1. Bacteria strains used in this study and their characteristics.**

| Strains | Phenotypes^b^ | Reference/Source |
| --- | --- | --- |
| Pathogenic bacterial strain | | |
| *Escherichia coli* CYCRE153 | Resistance to cefmetazole, cefotaxime, ceftazidime, cefepime, ertapenem, imipenem, meropenem, amikacin, and trimethoprim | Lab stock/clinical isolate |
| *Klebsiella pneumoniae* CYCRE114 | Carbapenem-resistant | Lab stock/clinical isolate |
| *Pseudomonas aeruginosa* CYCRE255 | Multidrug-resistant | Lab stock/clinical isolate |
| *Salmonella* Choleraesuis OU7526 | Multidrug-resistant | [5] |
| *Enterococcus faecium* Ef-4 | Resistance to vancomycin, amikacin, ciprofloxacin, levofloxacin, erythromycin, and gentamicin | Lab stock/clinical isolate |
| *Staphylococcus aureus* SA-1 | Resistance to methicillin, ciprofloxacin, and levofloxacin | Lab stock/clinical isolate |
| Probiotic bacterial strain^a^ |  |  |
| *Lactiplantibacillus pentosus* SLC13 | Experimental control strain | [1] |
| *Weissella confusa* CYLB30 | Amikacin-, ciprofloxacin-, gentamicin-, levofloxacin-, and metronidazole-resistant; EPS: 380.1 ± 7.2 mg/L | Beitou Market |
| *Pediococcus stilesii* CYLB33 | Amikacin-, ciprofloxacin-, gentamicin-, levofloxacin-, and metronidazole-resistant; EPS: 355.5 ± 9.9 mg/L | Beitou Market |
| *Lactobacillus futsaii* CYLB45 | Amikacin-, ciprofloxacin-, gentamicin-, levofloxacin-, and metronidazole-resistant; EPS: 378.49 ± 4.6 mg/L | Yongchun Market |
| *Lactiplantibacillus plantarum* CYLB47 | Cefazolin-, ciprofloxacin-, gentamicin-, levofloxacin-, and metronidazole-resistant; EPS: 368.15 ± 3.1 mg/L | Yongchun Market |
| *Weissella confusa* CYLB51 | Amikacin-, ciprofloxacin-, gentamicin-, levofloxacin-, and metronidazole-resistant; EPS: 375.96 ± 4.0 mg/L | Beitou Market |
| *Limosilactobacillus fermentum* CYLB55 | Amikacin-, ciprofloxacin-, gentamicin-, levofloxacin-, and metronidazole-resistant; EPS: 373.92 ± 9.9 mg/L | Beitou Market |

^a^The species of LAB strains were determined by 16s rRNA-sequencing.

^b^Antibiotic susceptibility to amikacin, amoxicillin/clavulanate, ampicillin, cefazolin, ceftriaxone, ciprofloxacin, clarithromycin, gentamicin, levofloxacin, metronidazole, penicillin G, and tetracycline, of probiotic strains, was determined by disk diffusion.

EPS, exopolysaccharide.

**Table S2. The antibacterial activity of isolated LAB strains against six pathogenic bacteria by using overlay assay.**

|  | **LAB strains (inhibition zone (mm))** | | | | | |
| --- | --- | --- | --- | --- | --- | --- |
| **Pathogenic bacteria** | CYLB30 | CYLB33 | CYLB45 | CYLB47 | CYLB51 | CYLB55 |
| *Escherichia coli* | 8 | 0 | 8 | 9 | 9 | 5 |
| *Klebsiella pneumoniae* | 6 | 0 | 0 | 6 | 0 | 3 |
| *Salmonella* Choleraesuis | 9 | 10 | 10 | 10 | 9 | 8 |
| *Pseudomonas aeruginosa* | 4 | 0 | 4 | 4 | 4 | 4 |
| *Enterococcus faecium* | 6 | 5 | 4 | 7 | 5 | 4 |
| *Staphylococcus aureus* | 6 | 6 | 3 | 7 | 5 | 3 |

| **Table S3. Characteristics of the chromosome and plasmids in CYLB30, CYLB47, and CYLB55.** | | | | | | | |
| --- | --- | --- | --- | --- | --- | --- | --- |
| **Genome** | **Genome characteristics** | | | | | | |
|  | **Size (bp)** | **Coverage** | **GC contents** | **Accession number** | **Number of coding sequences** | **Number of RNAs** | |
| **Chromosome** |  |  |  |  |  |  | |
| CYLB30 | 2,410,104 | 68 | 44.80 | CP120516 | 2,269 | 115 | |
| **Putative plasmids** |  |  |  |  |  |  | |
| pCYLB30-1 | 25,018 | 111 | 38.05 | CP120520 | 27 | - | |
| pCYLB30-2 | 23,195 | 31 | 34.15 | CP120518 | 35 | - | |
| pCYLB30-3 | 15,890 | 48 | 37.74 | CP120517 | 23 | - | |
| pCYLB30-4 | 13,579 | 152 | 39.75 | CP120519 | 13 | - | |
| pCYLB30-5 | 12,851 | 65 | 40.88 | CP120514 | 10 | - | |
| pCYLB30-6 | 10,284 | 33 | 40.83 | CP120515 | 11 | - | |
| **Chromosome** |  |  |  |  |  |  | |
| CYLB47 | 3,157,658 | 31 | 44.67 | CP120660 | 3,155 | 90 | |
| **Putative plasmids** |  |  |  |  |  |  |  |
| pCYLB47-1 | 71,871 | 57 | 41.61 | CP120659 | 77 | - | |
| pCYLB47-2 | 51,648 | 22 | 37.54 | CP120661 | 60 | - | |
| pCYLB47-3 | 49,839 | 41 | 39.87 | CP120656 | 58 | - | |
| pCYLB47-6 | 28,697 | 45 | 38.19 | CP120664 | 31 | - | |
| pCYLB47-7 | 28,179 | 20 | 37.99 | CP120663 | 39 | - | |
| pCYLB47-8 | 18,793 | 32 | 38.30 | CP120662 | 22 | - | |
| pCYLB47-9 | 15,893 | 20 | 33.49 | CP120665 | 21 | - | |
| pCYLB47-10 | 13,677 | 18 | 38.06 | CP120667 | 16 | - | |
| pCYLB47-11 | 11,629 | 20 | 40.00 | CP120666 | 10 | - | |
| **Chromosome** |  |  |  |  |  |  | |
| CYLB55 | 2,093,653 | 292 | 42.55 | CP120668 | 1,978 | 76 | |
| **Putative plasmids** |  |  |  |  |  |  | |
| pCYLB55-1 | 38,056 | 175 | 36.39 | CP120669 | 35 | - | |
| pCYLB55-2 | 8,672 | 47 | 31.59 | CP120670 | 8 | - | |

**Figure S1.**


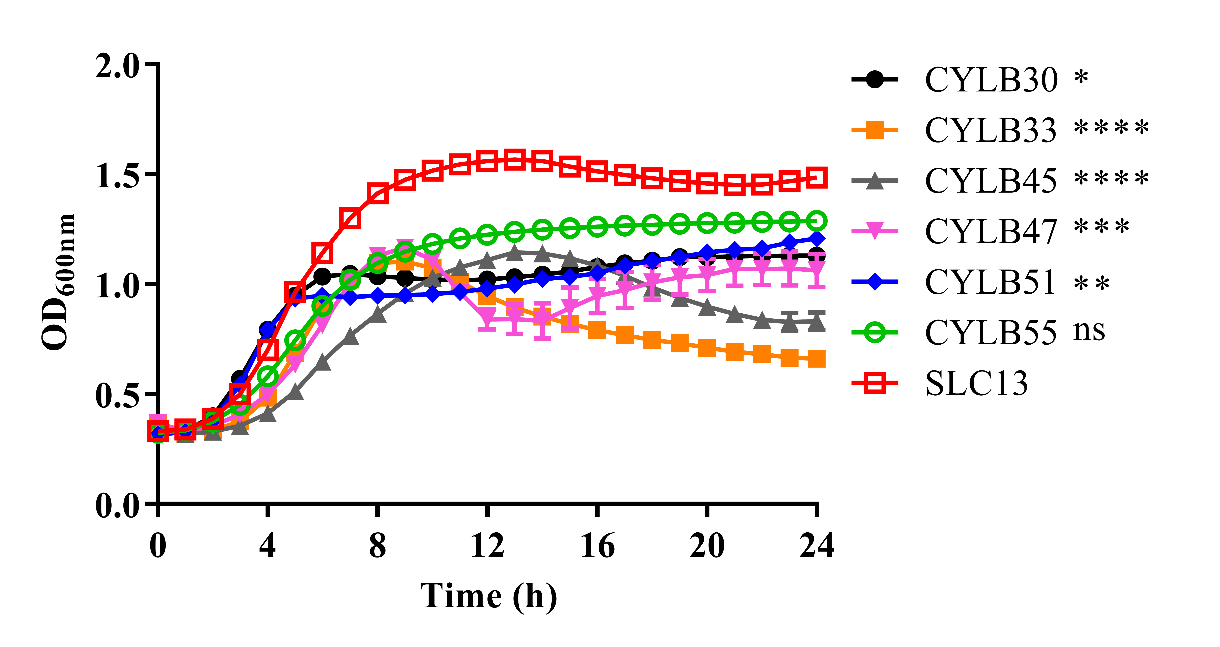


**Fig S1. Growth curve of isolated LAB strains incubated for 24 h in MRS broth (pH 6.5).** The growth rate of LAB isolated from this study was compared to SLC13. *, *p* < 0.05; **, *p* < 0.01; ***, *p* < 0.001; ****, *p* < 0.0001; ns, no significant difference.

**Figure S2**

| **(A).**  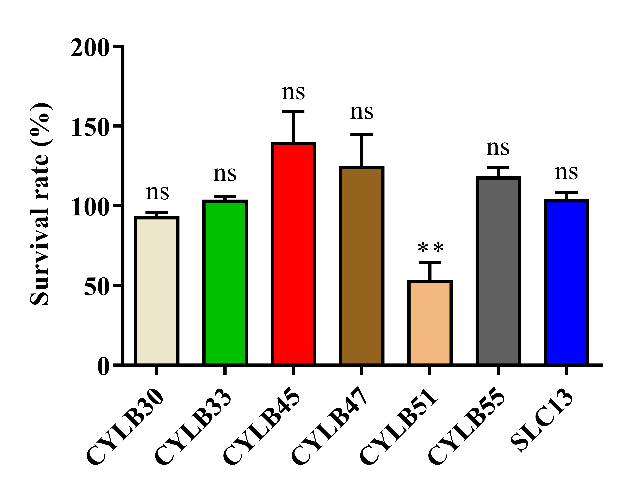 | **(B).**  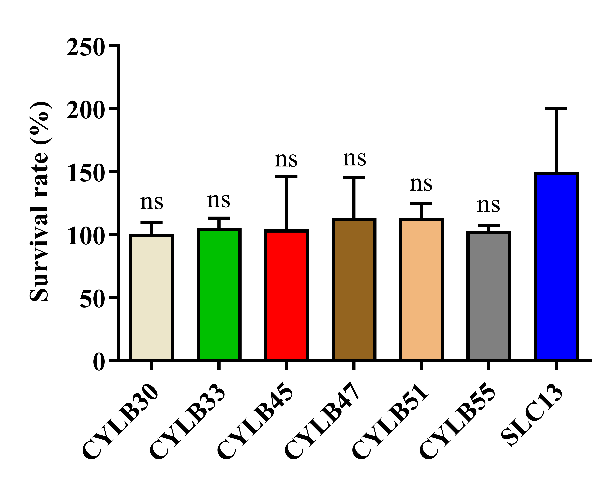 |
| --- | --- |

**Fig. S2.** The survival rate of LAB strains after incubation in acidic MRS broth (pH 3.0) for 3 h **(A)** and MRS broth with 0.3% bile salt (pH 6.5) for 4 h **(B)**. Error bars represent the standard deviation of triplicates. The survival rate of LAB isolated from this study in acidic MRS broth and MRS broth with 0.3% bile salt was compared to SLC13 under these culture conditions. **, *p* < 0.01; ns, no significant difference.

**Figure S3**


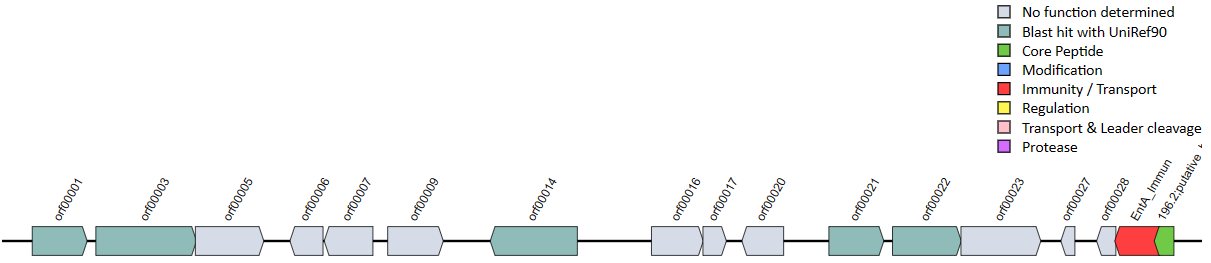


**Fig. S3.** Linear representation of anticipated lantibiotic gene clusters in *Lactiplantibacillus plantarum* CYLB47. The diagram, drawn approximately to scale, is based on information derived from genome sequences. Genes encoding products that are expected to resemble known lantibiotic-associated proteins are depicted in gray, while putative structural peptides are highlighted in green.

**Figure S4**


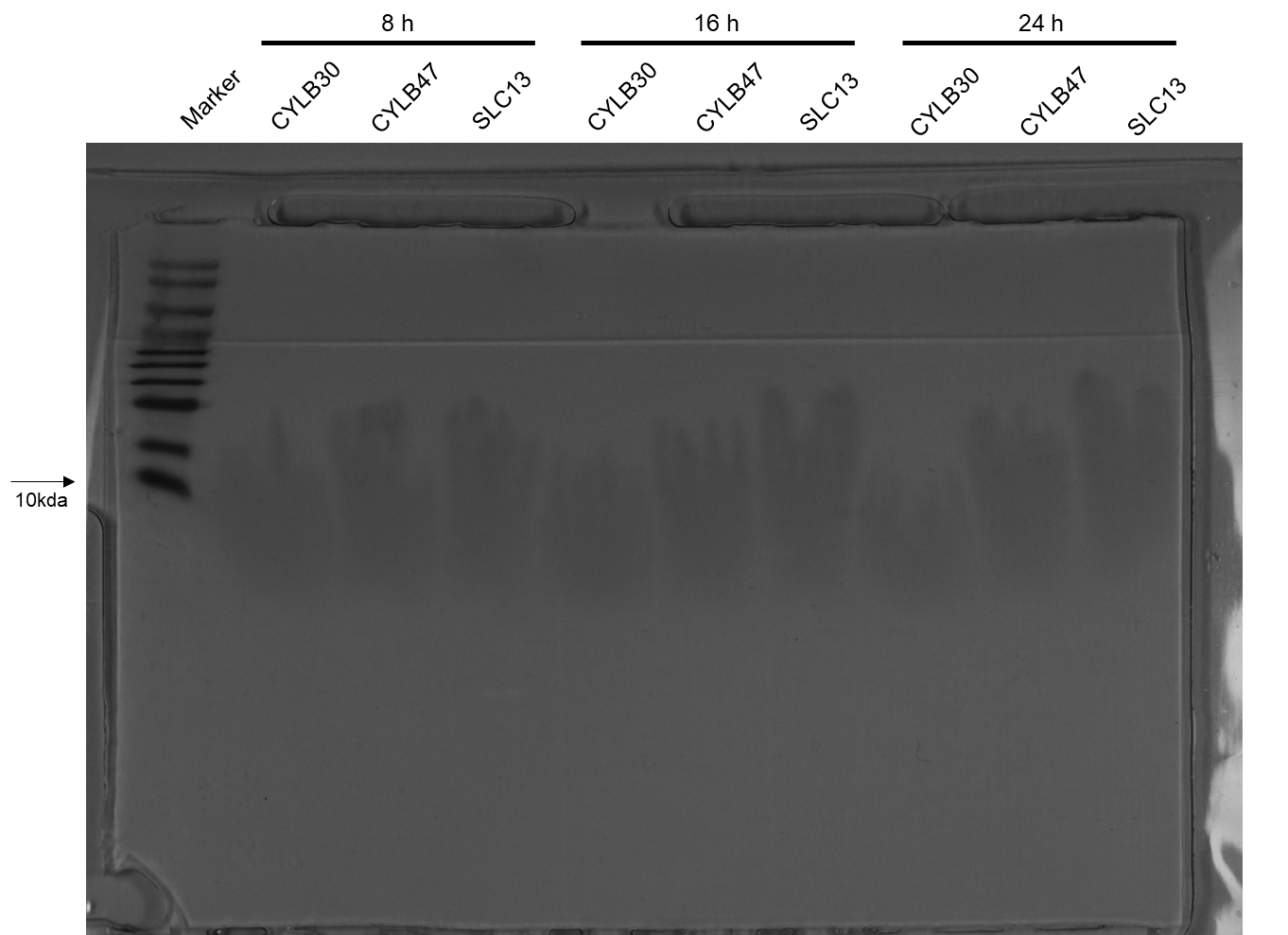


**Fig. S4.** Separation of bacteriocin-like inhibitory substance (BLIS) extracted from CYLB33, CYLB47, and SLC13 by SDS-PAGE. BLIS extracted from SLC13 was considered as positive control. The marker was PageRuler™ Prestained Protein Ladder (Thermo Scientific™) with ranged from 10-180kDa.

**Figure S5**

**(A).**


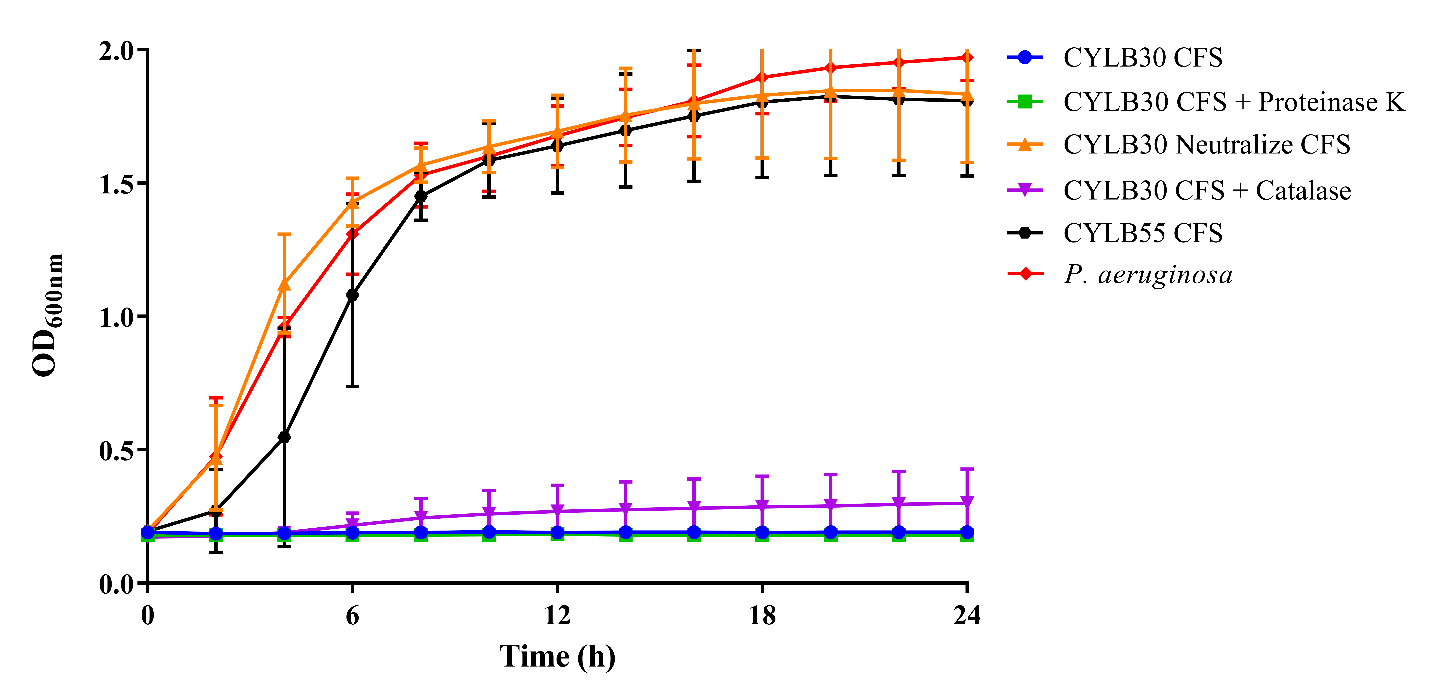


**(B).**


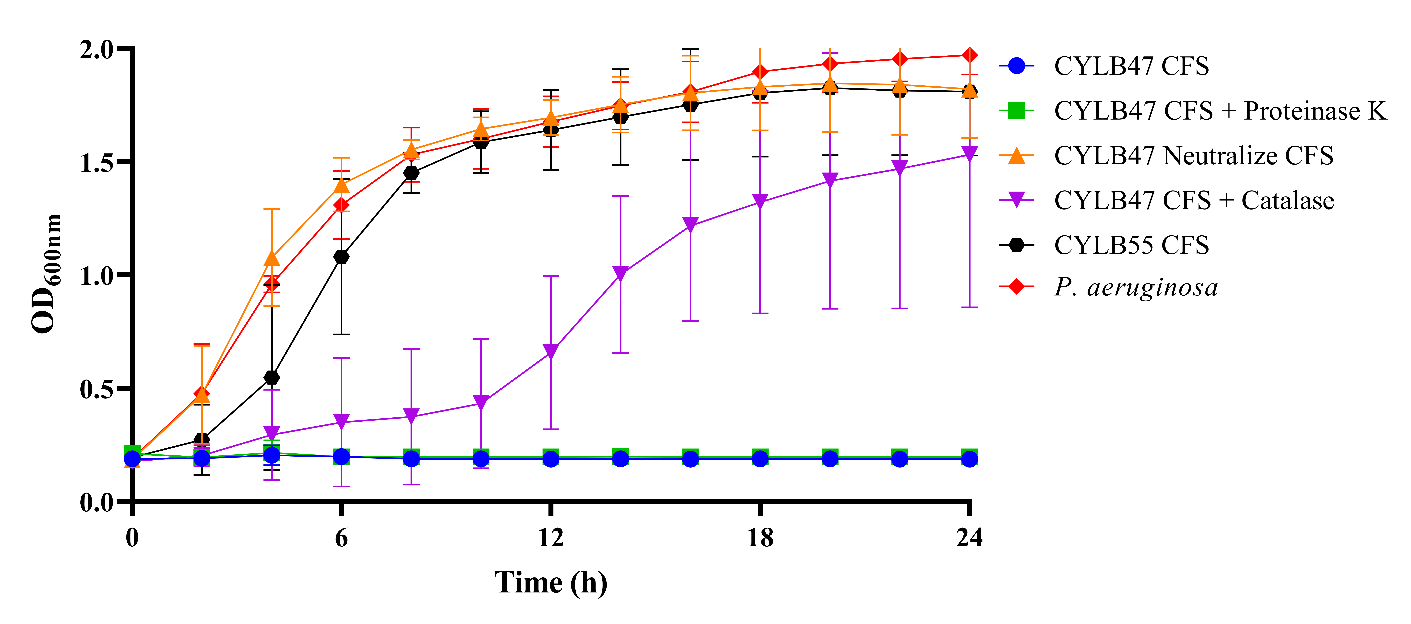


**Fig. S5.** Anti-*Pseudomonas aeruginosa* activity of cell-free supernatant derived from CYLB30 (A) and CYLB47 (B) after pH adjustment, proteinase K, and catalase treatment. CYLB55 CFS was used as a negative control.

**References**

1. Thuy TTD, Kuo P-Y, Lin S-M, Kao C-YJBm (2022). Anti-Helicobacter pylori activity of potential probiotic Lactiplantibacillus pentosus SLC13. 22(1):277.

2. Wen LL, Kuo PY, Thuy TTD, Duong TTT, Huang YT, Hsueh PR, Chen YC, Kao CY (2023). Genome-based characterization of conjugative IncHI1B plasmid carrying carbapenemase genes bla(VIM-1), bla(IMP-23), and truncated bla(OXA-256)in Klebsiella pneumoniae NTU107224. Infect Genet Evol 110:105420; doi: 10.1016/j.meegid.2023.105420.

3. Wick RR, Holt KE (2019). Benchmarking of long-read assemblers for prokaryote whole genome sequencing. F1000Res 8:2138; doi: 10.12688/f1000research.21782.4.

4. van Heel AJ, de Jong A, Song C, Viel JH, Kok J, Kuipers OP (2018). BAGEL4: a user-friendly web server to thoroughly mine RiPPs and bacteriocins. Nucleic Acids Res 46(W1):W278-W81; doi: 10.1093/nar/gky383.

5. Chiu L-H, Chiu C-H, Horn Y-M, Chiou C-S, Lee C-Y, Yeh C-M, Yu C-Y, Wu C-P, Chang C-C, Chu CJBm (2010). Characterization of 13 multi-drug resistant Salmonella serovars from different broiler chickens associated with those of human isolates. 10:1-10.
